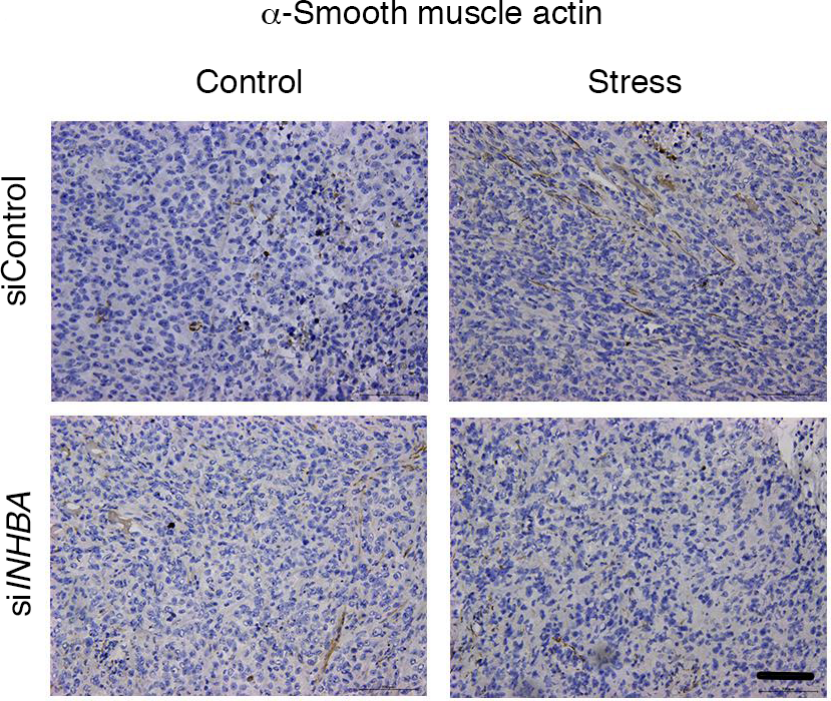# Adrenergic-mediated increases in *INHBA* drive CAF phenotype and collagens

**DOI:** 10.1172/jci.insight.149895

**Published:** 2021-04-08

**Authors:** Archana S. Nagaraja, Robert L. Dood, Guillermo Armaiz-Pena, Yu Kang, Sherry Y. Wu, Julie K. Allen, Nicholas B. Jennings, Lingegowda S. Mangala, Sunila Pradeep, Yasmin Lyons, Monika Haemmerle, Kshipra M. Gharpure, Nouara C. Sadaoui, Cristian Rodriguez-Aguayo, Cristina Ivan, Ying Wang, Keith Baggerly, Prahlad Ram, Gabriel Lopez-Berestein, Jinsong Liu, Samuel C. Mok, Lorenzo Cohen, Susan K. Lutgendorf, Steve W. Cole, Anil K. Sood

Original citation: *JCI Insight*. 2017;2(16):e93076. https://doi.org/10.1172/jci.insight.93076

Citation for this corrigendum: *JCI Insight*. 2021;6(7):e149895. https://doi.org/10.1172/jci.insight.149895

The Editors previously posted an Expression of Concern for this article regarding images in Figure 5E that appeared to be the same (siControl Stress and si*NHBA* Control). An institutional review committee concluded that the error in Figure 5E was inadvertent and recommended correction. The authors provided the correct image for the siControl Stress sample from the original study. The correct version of Figure 5E appears below.

The authors regret the error.

## Figures and Tables

**Figure 5:**